# The FACT subunit TbSpt16 is involved in cell cycle specific control of *VSG* expression sites in *Trypanosoma brucei*

**DOI:** 10.1111/j.1365-2958.2010.07350.x

**Published:** 2010-10

**Authors:** Viola Denninger, Alexander Fullbrook, Mohamed Bessat, Klaus Ersfeld, Gloria Rudenko

**Affiliations:** 1 Division of Cell and Molecular Biology, Sir Alexander Fleming Building, Imperial CollegeSouth Kensington, London SW7 2AZ, UK; 2 Department of Biological Sciences and Hull York Medical School, University of HullCottingham Road, Hull HU6 7RX, UK

## Abstract

The African trypanosome *Trypanosoma brucei* monoallelically expresses one of more than 1000 Variant Surface Glycoprotein (*VSG*) genes. The active *VSG* is transcribed from one of about 15 telomeric *VSG* expression sites (ESs). It is unclear how monoallelic expression of VSG is controlled, and how inactive *VSG* ESs are silenced. Here, we show that blocking synthesis of the *T. brucei* FACT subunit TbSpt16 triggers a G2/early M phase cell cycle arrest in both bloodstream and insect form *T. brucei*. Segregation of *T. brucei* minichromosomes in these stalled cells is impaired, implicating FACT in maintenance of centromeres. Strikingly, knock-down of TbSpt16 results in 20- to 23-fold derepression of silent *VSG* ES promoters in bloodstream form *T. brucei*, with derepression specific to the G2/M cell cycle stage. In insect form *T. brucei* TbSpt16 knock-down results in 16- to 25-fold *VSG* ES derepression. Using chromatin immunoprecipitation (ChIP), TbSpt16 was found to be particularly enriched at the promoter region of silent but not active *VSG* ESs in bloodstream form *T. brucei*. The chromatin remodeler FACT is therefore implicated in maintenance of repressed chromatin present at silent *VSG* ES promoters, but is also essential for chromosome segregation presumably through maintenance of functional centromeres.

## Introduction

The African trypanosome *Trypanosoma brucei* is a protozoan parasite, which causes African sleeping sickness in humans and ‘nagana’ in livestock, diseases that have a devastating impact on sub-Saharan Africa (Hotez and Kamath, 2009). *T. brucei* is a unicellular eukaryote which has branched off from the eukaryotic lineage relatively early (Sogin and Silberman, 1998; Embley and Martin, 2006), resulting in aspects of its molecular biology having atypical features compared with other eukaryotes. For example, transcriptional control in *T. brucei* is highly unusual. The *T. brucei* genome is organized in extensive polycistronic transcription units containing large arrays of unrelated genes, which are constitutively transcribed by RNA polymerase II (Pol II) (Berriman *et al*., 2005). Transcript levels are regulated post-transcriptionally. There does not appear to be significant regulation of Pol II transcription, even of the large number of genes which need to be expressed in a life cycle specific fashion as *T. brucei* differentiates into different life cycle stages as it cycles between the tsetse fly and the mammalian bloodstream (Clayton, 2002; Lustig *et al*., 2007; Queiroz *et al*., 2009; Veitch *et al*., 2010).

Another highly unusual feature of African trypanosomes is that RNA polymerase I (Pol I) does not exclusively transcribe ribosomal DNA (rDNA) as in other eukaryotes, but has also been recruited for transcription of a small subset of its protein coding genes (Gunzl *et al*., 2003). These Pol I-transcribed protein coding genes are subject to transcriptional control, making them the exception within the trypanosome genome. One of these is the Variant Surface Glycoprotein (*VSG*) gene encoding the major surface coat of bloodstream form *T. brucei*. Trypanosomes have developed a sophisticated strategy of antigenic variation of VSGs, allowing them to evade the host antibody response (Taylor and Rudenko, 2006; Morrison *et al*., 2009). A single trypanosome contains more than 1000 *VSG* genes, only one of which is expressed at a time from one of about 15 similar telomeric *VSG* expression sites (ESs) (Berriman *et al*., 2002; Hertz-Fowler *et al*., 2008). For antigenic variation to work, it is essential that only one *VSG* ES is active at a time, and it is still mysterious exactly how the ‘counting’ machinery behind this monoallelic transcription of *VSG* ESs operates (Figueiredo *et al*., 2009).

It has previously been argued that chromatin remodelling plays a role in silencing *VSG* ESs in insect form *T. brucei*; however, the situation in bloodstream form *T. brucei* was less clear (Navarro *et al*., 1999). Recently however, it has been shown that the chromatin structure of *VSG* ESs in bloodstream form *T. brucei* differs according to their activation state, where the active *VSG* ES is highly depleted of nucleosomes (Figueiredo and Cross, 2010; Stanne and Rudenko, 2010). This indicates that chromatin remodelling plays a key role in *VSG* ES regulation. In agreement with this, several proteins involved in the epigenetic control of transcription have been shown to be involved in downregulation of *VSG* ESs. These include TbISWI, a member of the SWI2/SNF2 superfamily of ATP-dependent chromatin remodelling proteins, the telomere binding protein RAP1 and the histone methyltransferase DOT1 (Hughes *et al*., 2007; Figueiredo *et al*., 2008; Yang *et al*., 2009).

As chromatin remodelling is clearly of critical importance for *VSG* ES control, we investigated the role of the FACT (facilitates chromatin transcription) chromatin remodelling complex, which, unlike ISWI, does not require ATP hydrolysis for nucleosome remodelling. The FACT complex is a heterodimer of the Spt16 and SSRP1 proteins in humans (Orphanides *et al*., 1999), or Spt16 and the subunits Pob3 and Nhp6 in *S. cerevisiae* (VanDemark *et al*., 2006). The FACT complex functions as a histone chaperone. FACT can deposit core histones onto DNA, or can displace one H2A-H2B dimer from the nucleosome thereby facilitating transcription (Belotserkovskaya *et al*., 2003; reviewed in: Belotserkovskaya *et al*., 2004; Reinberg and Sims 3rd, 2006; Krebs and Tora, 2009). FACT has been shown to play a role in transcription elongation (Belotserkovskaya *et al*., 2003), and in suppressing initiation of inappropriate transcription from cryptic promoters (Mason and Struhl, 2003). In addition, FACT plays a role in chromosome segregation through its function in the maintenance of centromeric heterochromatin (Lejeune *et al*., 2007; Okada *et al*., 2009).

Here, we show that blocking synthesis of the FACT subunit Spt16 in *T. brucei* triggers a G2/early M cell cycle arrest, with disruption of minichromosome segregation and cells unable to complete mitosis. Knock-down of TbSpt16 also results in derepression of *VSG* ES promoters in both bloodstream and insect form *T. brucei*, but not to an increase in *VSG* ES switching. Strikingly, the observed *VSG* ES derepression in bloodstream form *T. brucei* is specific to cells arrested in the G2/early M cell cycle stage. This indicates that components of the FACT complex are not only involved in maintaining the chromatin structure necessary for inactivation of the silent *VSG* ESs, but also contribute to proper chromosome segregation.

## Results

### Knock-down of TbSpt16 in bloodstream form *T. brucei* leads to a precise cell cycle arrest

**Fig. 1:**
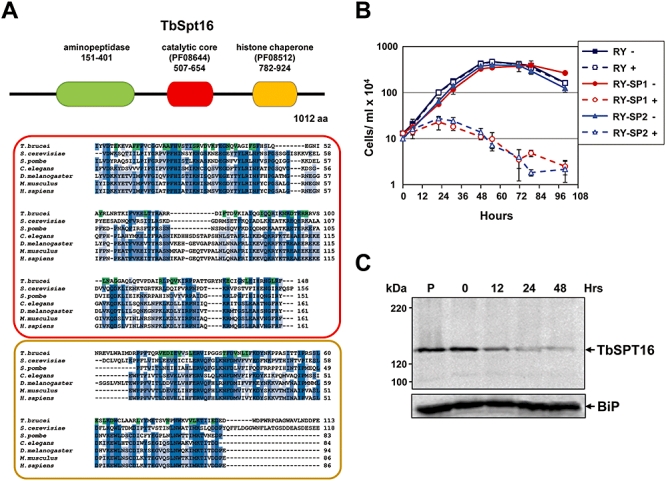
*T. brucei* TbSpt16 is an orthologue of the FACT subunit Spt16, and is essential in bloodstream form *T. brucei*. A. Location of protein domains in TbSpt16 (Accession No. Tb927.3.5620) as annotated using SMART. The aminopeptidase domain (*e*-value: 5.00 e^−11^), the central catalytic core (*e*-value: 7.20 e^−69^) and the histone chaperone domain (*e*-value: 2.40 e^−42^) are indicated. A ClustalW2 alignment of the Spt16 protein sequence over the catalytic core (red box) and the histone chaperone domain (yellow box) is shown with the *T. brucei* TbSpt16 sequence compared with the Spt16 orthologues from *S. cerevisiae*, *S. pombe*, *C. elegans*, *D. melanogaster*, *M. musculus* and *H. sapiens* (accession numbers listed in the *Experimental procedures*). Conserved amino acids are in blue (dark blue: 6–7 sequences conserved; mid-blue: 5 of 7 sequences conserved; light blue: 3–4 of 7 sequences conserved). Amino acids which are different in *T. brucei* but have conserved properties are indicated in green. B. TbSpt16 is essential in bloodstream form *T. brucei*. Tetracycline-inducible RNAi against TbSpt16 was induced in *T. brucei* RY-SP1 and RY-SP2. Growth curves are shown in the presence (+) or absence (−) of tetracycline compared with the parental *T. brucei* RY cell line. Cells were cultured and cell densities were determined until the parental cell line had reached stationary phase. The average of three measurements is plotted against time, with standard deviation indicated with error bars.C. Knock-down of TbSpt16 as confirmed using Western blotting. Protein lysates of *T. brucei* RY-SP1 after the induction of TbSpt16 RNAi for the time in hours (Hrs) were compared with protein lysate from the parental cell line (P). TbSpt16 and BiP (loading control) are indicated. Size markers in kiloDaltons (kDa) are indicated on the left.

**Fig. 2:**
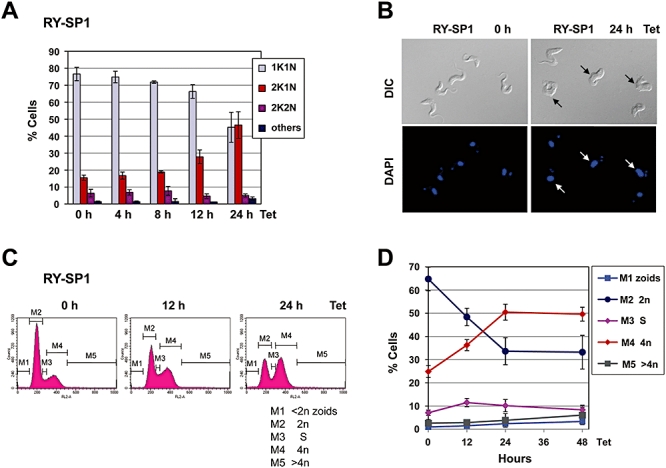
Inhibition of TbSpt16 synthesis in bloodstream form *T. brucei* affects mitosis, as induction of RNAi leads to an accumulation of stalled cells with fully replicated (4n) DNA content but with two kinetoplasts and one nucleus (2K1N). A. Analysis of the *T. brucei* cell cycle after the induction of TbSpt16 RNAi was performed using microscopic analysis of DAPI stained cells. Cells (n∼400) were categorized according to the numbers of nuclei (N) or kinetoplasts (K) present. Cells with unusual numbers of nuclei or kinetoplasts are combined in the category ‘others’. The percentage of cells in the different categories is plotted against time in hours (h) of induction of TbSpt16 RNAi using tetracycline (Tet). The average of three independent experiments is plotted with standard deviation indicated with error bars. B. Representative images of differential interference constrast (DIC) or DAPI-stained *T. brucei* RY-SP1 cells are shown after either 0 or 24 h induction of TbSpt16 RNAi with tetracycline (Tet). Accumulated 2K1N cells are indicated with arrows. C. Accumulation of cells with double DNA content (4n). Total DNA content of *T. brucei* RY-SP1 cells was determined using FACS analysis of propidium iodide stained cells after the induction of TbSpt16 RNAi with tetracycline (Tet) for the time indicated in hours (h). The number of cells fluorescing in the FL-2 channel (*x*-axis) at the different time points is plotted. The gates used are: M1 (< 2n DNA content or ‘zoids’), M2 (2n DNA content), M3 (S phase), M4 (4n) or M5 (> 4n). D. Blocking synthesis of TbSpt16 leads to an accumulation of cells with double DNA content (4n). The mean percentage of cells in different cell cycle stages as quantified using propidium iodide FACS is plotted against time in hours of tetracycline induction of TbSpt16 RNAi. The different gates used are as shown in (C). The values shown are the mean of three different experiments with the standard deviation indicated with error bars.

We identified the *T. brucei* orthologue of the Spt16 subunit of FACT (Accession No. Tb927.3.5620) on the basis of its similarity to Spt16 from mouse and yeast (Fig. 1A). This is consistent with previous investigations in *T. brucei* and *Leishmania major* where components of the FACT complex were identified (Martinez-Calvillo *et al*., 2007; Patrick *et al*., 2008). In order to investigate the role of FACT in bloodstream form *T. brucei*, we performed tetracycline-inducible RNA interference (RNAi) against TbSpt16. Two independent *T. brucei* clones RY-SP1 and RY-SP2 showed a rapid reduction in growth after 12 h induction of TbSpt16 RNAi (Fig. 1B) (Patrick *et al*., 2008). Western blot analysis confirmed successful knock-down of TbSpt16 protein to 5% wild-type levels after 48 h (Fig. 1C). Cells were analysed microscopically in order to determine at which point in the cell cycle the growth arrest was occurring. A G1 trypanosome contains one nucleus (N) and one kinetoplast (K), where the kinetoplast replicates and divides before the nucleus (2K1N cells), after which the nucleus undergoes mitosis (2K2N cells) (Woodward and Gull, 1990). Within 24 h induction of TbSpt16 RNAi, an accumulation of cells with two kinetoplasts and one nucleus (2K1N) is observed (Fig. 2A and B). The percentage of 2K1N cells increased to a maximum of about 45% of the population after 24 h induction, and remained constant until 48 h (Fig. 2A). This cell cycle arrest is different to the precytokinesis arrest observed after blocking VSG synthesis, which results in an accumulation of 2K2N cells (Sheader *et al*., 2005).

The DNA content of these stalled 2K1N cells was determined using FACS analysis on propidium iodide (PI) stained cells (Fig. 2C). In a normal population, most of the cells are in G1 with a 2n DNA content. As cells progress through S phase, DNA content increases until it reaches 4n. After induction of TbSpt16 RNAi for 24 h an increase in cells with a double DNA content (4n or gate M4) (Fig. 2C and D) to 55% of the population was observed, but no great increase in cells in S phase. This accumulation of cells with fully replicated genomes is compatible with TbSpt16 playing a primary role in chromosome segregation rather than DNA replication.

In order to confirm this, we investigated DNA replication using 5-bromo-2′-deoxyuridine (BrdU) incorporation, which can be visualized using an anti-BrdU antibody and immunofluorescence microscopy (Fig. S1). Representative cells in either 1K1N or 2K1N stages of the cell cycle are shown. DNA replication continues in cells after 12 h induction of TbSpt16 RNAi, despite the fact that at this time point half maximal cell cycle arrest with an accumulation of 2K1N cells is already observed. As a control, we incubated cells in 1 µM aphidicolin, resulting in a block in DNA replication and cells negative for BrdU incorporation (result not shown). Together, all of these data are compatible with a scenario whereby blocking TbSpt16 synthesis leads to accumulation of 2K1N cells with double DNA content (4n) with the primary restriction being on chromosome segregation rather than DNA replication.

### Inhibiting TbSpt16 synthesis results in disruption of chromosome segregation and a cell cycle block in mitosis

**Fig. 3:**
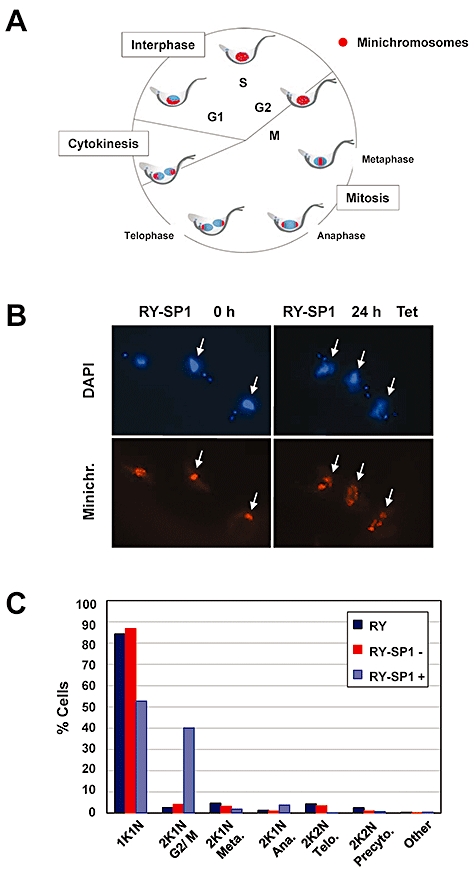
Blocking TbSpt16 synthesis in bloodstream form *T. brucei* leads to a disruption in chromosome segregation. A. Schematic of chromosome segregation during the cell cycle of bloodstream form *T. brucei* as adapted from Gull *et al*. (1998). Cells in G1-phase contain one nucleus with 2n DNA content (indicated with a large blue oval) with the minichromosomes arranged as a red crescent at the nuclear periphery. Entering S phase, first the kinetoplast DNA (small blue dot) and then the nuclear DNA are replicated resulting in a cell with 4n DNA content. In early G2 phase, first the kinetoplast divides (2K1N) and then the nucleus enters mitosis. Following assembly of the metaphase plate, the chromosomes are segregated and pulled to the different spindle poles by microtubules. The segregation of minichromosomes proceeds before segregation of the large chromosomes. Finally, the nucleus divides and the cell undergoes cytokinesis. B. Blocking TbSpt16 synthesis results in disruption of minichromosome segregation in bloodstream form *T. brucei*. Fluorescent *in situ* hybridization (FISH) experiments visualizing *T. brucei* minichromosomes (Minichr.), using biotin labelled 177 bp repeat probes, were performed in the presence or absence of tetracycline (Tet) for 24 h (h) to induce TbSpt16 RNAi. The top panel shows DAPI stained cells. The arrows indicate 2K1N cells, with minichromosomes assembled at the metaphase plate in the absence of TbSpt16 RNAi. After inducing a knock-down of TbSpt16, a large number of minichromosomes in 2K1N cells show minichromosomal clusters dispersed throughout the nucleus. C. Disruption of TbSpt16 synthesis results in an accumulation of cells in G2/M. Quantification of cell cycle distribution of cells after performing minichromosome FISH in *T. brucei* RY-SP1 in the presence (+) or absence (−) of tetracyline for 24 h to induce TbSpt16 RNAi. Results were compared with the parental cell line (RY). FISH was performed using a minichromosome specific 177 bp repeat probe, and cells were categorized into different stages of the cell cycle as indicated in (A): 1K1N, 2K1N G2/M with minichromosomes distributed over the nucleus, 2K1N metaphase (Meta.) with minichromosomes present at a metaphase plate, 2K1N anaphase (Ana.) with minichromosomes present at the poles of the nucleus, 2K2N telophase (Telo) with minichromosomes still found as a distinct dot in the nucleus, 2K2N precytokinesis (Precyto.) with minichromosomes still at the poles but a bit more diffuse or ‘others’ with unusual numbers of nuclei or kinetoplasts. Shown is the mean of two independent experiments.

In order to further investigate the impact of TbSpt16 on chromosome segregation, we performed fluorescent *in situ* hybridization (FISH) experiments. The *T. brucei* genome contains large megabase chromosomes as well as 100 transcriptionally inactive minichromosomes (Melville *et al*., 1998; 2000; Wickstead *et al*., 2004). A schematic diagram of chromosome segregation events during mitosis in bloodstream form *T. brucei* is shown in Fig. 3A. After replication of the kDNA and during replication of the nuclear DNA, the kinetoplast divides. After entry into mitosis, although chromosomes of *T. brucei* do not visibly condense, they congregate in the centre of the nucleus, equivalent to the metaphase plate configuration in mammalian cells. During anaphase, minichromosomes and with a short delay large chromosomes are segregated to opposite spindle poles (Ersfeld and Gull, 1997). During this process large chromosomes are attached to microtubules via the kinetochore/centromere complex. In contrast, the minichromosomes segregate through a different process, as they exceed the number of available spindle microtubules and do not contain centromeric sequences similar to those identified on large chromosomes (Ersfeld and Gull, 1997; Wickstead *et al*., 2004; Obado *et al*., 2007; Bessat and Ersfeld, 2009).

After blocking TbSpt16 synthesis for 24 h, FISH experiments revealed a disruption in minichromosome segregation in the stalled 2K1N cells in both bloodstream and procyclic form *T. brucei* (Figs 3B and S2A). Normally in 2K1N cells undergoing chromosome segregation, the segregating minichromosomes are present as a compact aggregation at metaphase. In contrast, in 2K1N cells stalled due to TbSpt16 RNAi, the minichromosomes were invariably visible as a diffuse spotty signal that was dispersed over the nucleus, indicating problems with chromosome congression and segregation. Quantification of these FISH experiments revealed that nearly all of these accumulated 2K1N cells had not entered metaphase but remained trapped in G2/early M phase (Figs 3C and S2B).

Segregation of large chromosomes can best be observed in cells during or shortly after the anaphase stage of mitosis, when the chromosomes are at the spindle poles (Bessat and Ersfeld, 2009). However, the cells stalled after TbSpt16 knock-down arrest very rapidly in G2/early M before anaphase is reached. As so few of these stalled cells reach the anaphase stage of mitosis, segregation of these larger chromosomes could not be adequately investigated.

### Blocking synthesis of TbSpt16 in insect form *T. brucei* results in arrested cells with an increase in zoids

**Fig. 4:**
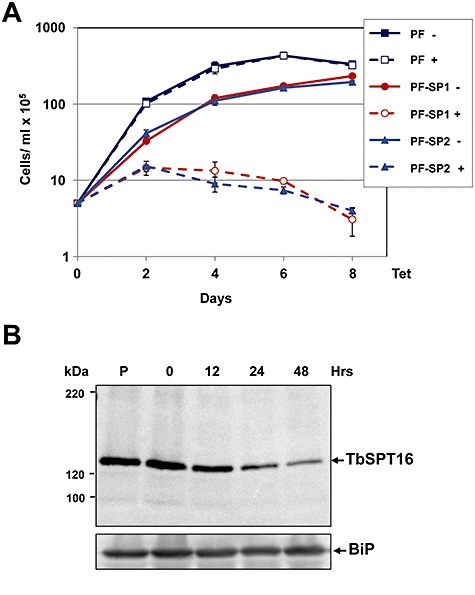
TbSpt16 is essential in insect form *T. brucei.* A. The growth of two independent procyclic form *T. brucei* Spt16 RNAi clones (PF-SP1 and PF-SP2) was monitored in the presence (+) or absence (−) of tetracycline (Tet) to induce TbSpt16 RNAi for the time indicated in days. In comparison the parental *T. brucei* 29–13 line is shown (PF). Cell lines were cultured and cell density was measured until the parental cell line had reached the stationary phase. B. Verification of successful knock-down of TbSpt16 by Western blotting. Protein lysates of *T. brucei* PF-SP1 were prepared after the induction of TbSpt16 RNAi for the time in hours (Hrs) indicated above. Protein lysate from the parental *T. brucei* 29–13 cell line (P) is shown for comparison. The blot was probed with an anti-TbSpt16 antibody or with anti-BiP as a loading control. Protein size markers are indicated on the left with sizes in kiloDaltons (kDa).

**Fig. 5:**
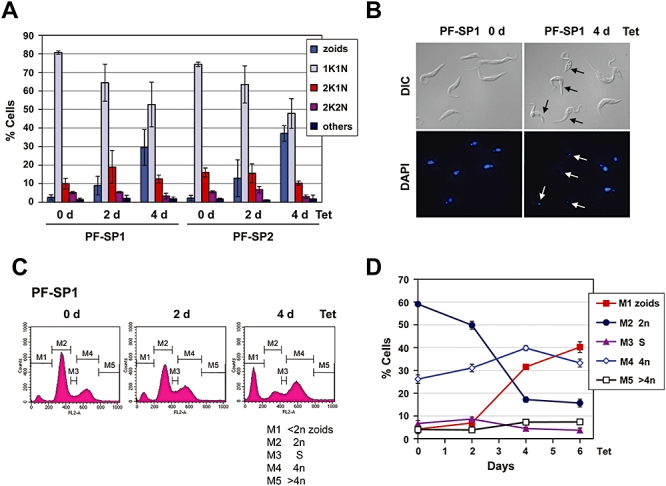
Blocking TbSpt16 synthesis in insect form *T. brucei* leads to an increase in zoids (1K0N cells with a DNA content of less than 2n). A. TbSpt16 RNAi was induced with tetracycline (Tet) in procyclic form *T. brucei* PF-SP1 or PF-SP2 cells for the time in days (d) indicated below. Cells were stained with DAPI, and categorized according to number of nuclei (N) or kinetoplasts (K). Zoids are 1K0N cells. Cells with unusual combinations of nuclei or kinetoplasts are indicated in ‘others’. The percentage of cells in the different categories is shown. Results are the mean of three independent experiments with the standard deviation indicated with error bars. B. Increase in zoids (1K0N) after the induction of TbSpt16 RNAi. Representative images of DIC or DAPI stained *T. brucei* PF-SP1 cells are shown after the induction of TbSpt16 RNAi with tetracycline (Tet) for 0 or 4 days (d). Representative examples of zoids (1K0N) are indicated with arrows. C. Blocking synthesis of TbSpt16 leads to an accumulation of zoids (cells with < 2n DNA content). DNA content of propidium iodide stained *T. brucei* PF-SP1 cells was determined by FACs after the induction of TbSpt16 RNAi for the time indicated. The degree of fluorescence in the FL-2 channel is indicated on the *x*-axis, with the gates used being: M1 (zoids, or DNA content < 2n), M2 (2n), M3 (S phase), M4 (4n), M5 (> 4n). D. Quantification of the increase in zoids as determined by FACs analysis of propidium iodide stained cells to measure DNA content. The different gates used are as shown in (C). TbSpt16 RNAi was induced, and the percentage of cells within the different gates is plotted, with the mean of three experiments shown (standard deviation indicated with error bars).

Procyclic form *T. brucei* shows significant differences from the bloodstream form in various molecular features including chromatin structure (Schlimme *et al*., 1993). As observed in the bloodstream form, knock-down of TbSpt16 in procyclic form *T. brucei* PF-SP1 and PF-SP2 leads to an arrest in cell growth after 2 days (Fig. 4A). There was efficient knock-down of the TbSpt16 protein, with only 13% wild-type levels remaining after 48 h induction of RNAi (Fig. 4B). Microscopic analysis of these stalled cells showed only a minor increase in the percentage of 2K1N cells within the stalled population. However, there was a dramatic increase in the percentage of zoids (1K0N cells), which reached 30–40% of the population by 4 days (Fig. 5A). Zoids are cells containing a single kinetoplast but no nucleus (1K0N) (Ploubidou *et al*., 1999). This cell type is more frequently observed in knock-down experiments in procyclic form rather than bloodstream form *T. brucei*, as the cell cycle checkpoints differ between these two life cycle stages (Ploubidou *et al*., 1999; Hammarton *et al*., 2003). In bloodstream form *T. brucei* a block in nuclear division also leads to a block in cell division, resulting in 2K1N cells. In contrast, procyclic form *T. brucei* are able to complete cytokinesis even after failed karyokinesis, and in the face of a block in nuclear division, cells still divide, producing both a 1K1N cell with 4n DNA content and a zoid (1K0N) (Gluenz *et al*., 2008).

After blocking TbSpt16 synthesis in procyclic *T. brucei*, microscopic analysis showed accumulation of zoids (Fig. 5B). DNA content of these stalled cells was determined using PI staining and FACS. These experiments confirm that there is an increase in zoids with < 2n DNA content from 5% of the population in uninduced cells to about 40% of the population after the induction of TbSpt16 RNAi for 4 days (Fig. 5C and D). Consistent with our bloodstream form results, there is an increase in cells with 4n DNA content, indicating that DNA replication is unaffected. In summary, blocking TbSpt16 synthesis triggers an arrest in nuclear division in both *T. brucei* life cycle stages. Due to differences in cell cycle progression, this leads to either an accumulation of 2K1N cells in bloodstream form *T. brucei* or an accumulation of 1K0N cells in procyclic form *T. brucei*.

### Inhibition of TbSpt16 synthesis leads to derepression of *VSG* ESs in both bloodstream and insect form *T. brucei*

**Fig. 6:**
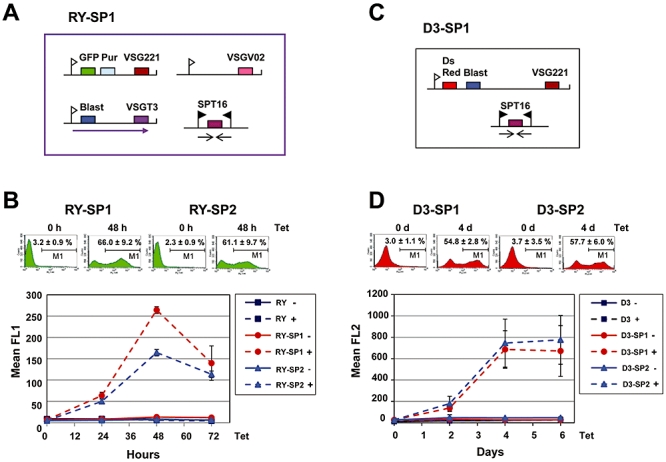
Blocking TbSpt16 synthesis results in derepression of silent VSG ESs in both bloodstream and insect form *T. brucei*. A. Schematic of the bloodstream form *T. brucei* reporter line RY-SP1. A blasticidin resistance gene (Blast) is inserted in the active *VSGT3* ES (transcription indicated with a violet arrow). The inactive *VSG221* ES has *eGFP* and a puromycin resistance gene (Pur) inserted immediately downstream of the promoter. After the induction of TbSpt16 RNAi from opposing tetracycline-inducible T7 promoters (black flags) derepression of *eGFP* can be monitored by FACS. B. Blocking synthesis of TbSpt16 by the induction of TbSpt16 RNAi results in derepression of *VSG* ESs in bloodstream form *T. brucei*. *T. brucei* RY-SP1 and RY-SP2 were incubated in the presence (+) or absence (−) of tetracycline to induce TbSpt16 RNAi, and cells were monitored for fluorescence in the FL1 channel. Cell fluorescence indicates derepression of the *eGFP* gene present in the silent *VSG221* ES. Representative FACS traces are shown with the percentage of cells in the M1 gate indicated above. The graph shows the total degree of fluorescence in the FL1 channel plotted against time. Results are the mean of three independent experiments, with standard deviation indicated with error bars. C. Schematic of the procyclic *T. brucei* D3-SP1 reporter line. A DsRed gene and a blasticidin resistance gene (Blast) are inserted behind the *VSG221* ES promoter (white flag). TbSpt16 RNAi can be induced from opposing tetracycline-inducible T7 promoters (black flags) and derepression of *DsRed* can be monitored by FACS. D. Induction of TbSpt16 RNAi results in *VSG* ES derepression in procyclic form *T. brucei*. *T. brucei* D3-SP1 and D3-SP2 cells were incubated in the presence (+) or absence (−) of tetracycline to induce TbSpt16 RNAi, and *VSG* ES promoter derepression was monitored by FACS in the FL2 channel. Representative FACS traces are shown above, with the percentage of cells present in the M1 gate indicated above. The graph shows the total degree of fluorescence in the FL2 channel over time, with the results plotted showing the mean of three independent experiments with standard deviation indicated with error bars.

A major role of FACT is facilitating transcription of RNA polymerase I, II and III transcription units through nucleosome remodelling (Birch *et al*., 2009). In order to investigate whether FACT could be involved in the chromatin remodelling associated with *VSG* ES control, we monitored *VSG* ES derepression in a bloodstream form *T. brucei* reporter cell line with an *eGFP* gene inserted downstream of the promoter of a silent *VSG221* ES (Fig. 6A) (Hughes *et al*., 2007). Following induction of TbSpt16 RNAi, *eGFP* derepression was monitored by FACS. After 48 h of RNAi, more than 60% of the population showed significant derepression of the silent *VSG221* ES (measured in the M1 gate indicated in Fig. 6B). Levels of *VSG221* ES derepression were 20- to 23-fold background after the induction of TbSpt16 RNAi for 48 h.

In order to investigate the role of TbSpt16 in *VSG* ES silencing in procyclic form *T. brucei*, we induced TbSpt16 RNAi in the *T. brucei* D3 reporter cell line containing a *DsRed* gene inserted downstream of the silent *VSG221* ES promoter (Fig. 6C) (Hughes *et al*., 2007). Derepression of the *DsRed* gene in *T. brucei* D3-SP1 and D3-SP2 was monitored by FACS. Induction of TbSpt16 RNAi resulted in *VSG* ES derepression in more than 50% of the cells (gate M1 in Fig. 6D), where total levels of fluorescence reached more than 16- to 25-fold background after 4 days.

**Fig. 7:**
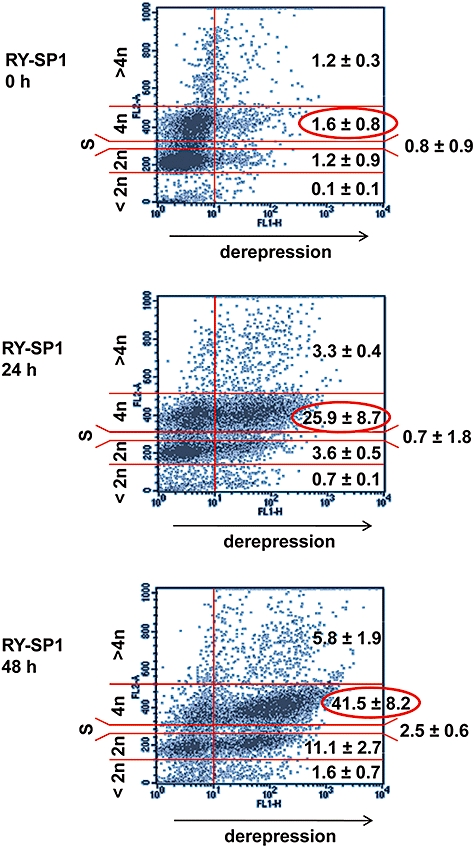
The *VSG* ES derepression observed in bloodstream form *T. brucei* after the induction of a block in TbSpt16 synthesis, is mainly present in cells in the G2/M stage (4n DNA content) of the cell cycle. TbSpt16 RNAi was induced in bloodstream form *T. brucei* RY-SP1 for the time indicated in hours (h). Derepression of the *VSG* ES located *eGFP* gene was monitored in the FL1 channel (*x*-axis). Cells with fluorescence levels in the FL1 channel above 20 are considered derepressed (right of the vertical red line). The propidium iodide-stained DNA content of the cells was simultaneously monitored in the FL2 channel (*y*-axis), where the horizontal red lines subdivide cell populations with DNA contents of, respectively, < 2n, 2n, S, 4n or > 4n. Within the boxes are shown the percentage of derepressed cells in different stages of the cell cycle. The percentage of derepressed cells with a 4n DNA content is indicated with a red oval. A representative experiment is shown, with the values presented being the mean of three independent experiments with the standard deviation indicated.

Intriguingly, in both *T. brucei* life cycle stages, derepression of *VSG* ESs was not observed in the entire population, as was seen after blocking TbISWI synthesis (Fig. S3) (Hughes *et al*., 2007). In contrast, FACS analysis showed a bimodal distribution, indicating that *VSG* ES promoter derepression was occurring in a subpopulation. In order to investigate a possible link between cell cycle arrest and *VSG* ES derepression, levels of eGFP were monitored in cells where DNA content was simultaneously analysed with PI staining using FACS analysis (Fig. 7). The most dramatic change in *VSG* ES derepression was observed in cells with 4n DNA content in the G2/M cell cycle stage. Cells with 4n DNA content and significant *VSG* ES derepression increased from 1.6% of the population to about 26% after 24 h induction of TbSpt16 RNAi and to 42% of the population after 48 h induction of RNAi. This increase in FL1 fluorescence is not simply a consequence of the average increase in cell size in the G2/M stage, as cells with eGFP inserted in the active *VSG* ES do not shift to higher fluorescence values after the induction of TbSpt16 RNAi (Fig. S3). The overall distribution of cells in the G1, S or G2/M phases was comparable to data in Fig. 2C, and showed an increase in cells in the G2/M phase from 28% to 55% total after the induction of TbSpt16 RNAi for 48 h. Therefore, the *VSG* ES derepression observed after the knock-down of TbSpt16 appears to be strongly correlated with the G2/early M phase of the cell cycle.

### Disruption of TbSpt16 leads to derepression of *VSG* ES promoters but not to significantly increased *VSG* ES switching

**Fig. 8:**
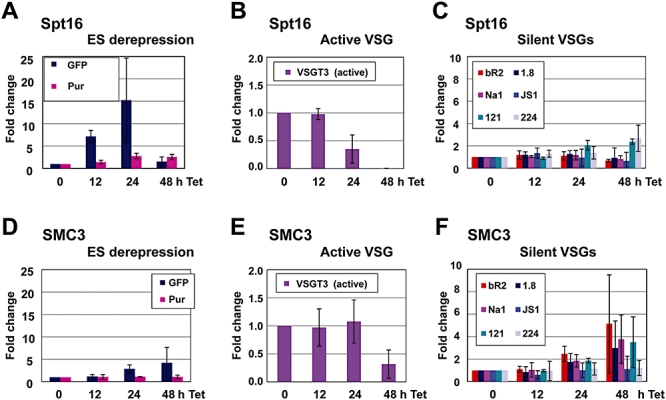
Blocking TbSpt16 synthesis in bloodstream form *T. brucei* results in derepression of silent *VSG* ES promoters, but no significant increase in *VSG* ES switching. In contrast, knock-down of the cohesin subunit SMC3 does not lead to significant derepression of silent *VSG* ES promoters, but does result in an increase in *VSG* ES switching (Landeira *et al*., 2009). Transcript amounts were quantified using qPCR. Transcript levels at the beginning of each time-course were set at 1, and results were plotted as fold change. Results shown are the mean of three independent experiments with the standard deviation indicated with error bars. A. Derepression of *VSG* ESs after induction of TbSpt16 RNAi for the time indicated in hours (h), as monitored through quantification of *eGFP* and puromycin (Pur) transcript from the derepressed *VSG221* ES. B. Decrease in *VSGT3* transcript from the active *VSGT3* ES after knock-down of TbSpt16. C. Analysis of VSG switching through monitoring *VSG* transcripts from silent *VSG* ESs. The bloodstream form *T. brucei* RY-SP1 strain had TbSpt16 RNAi induced for the time in hours (h) indicated. The amount of different *VSG* transcripts is plotted, whereby the *VSGbR2, VSG1.8, VSGNA1, VSGJS1, VSG121* and *VSG224* are all in silent *VSG* ESs. D. Quantification of *eGFP* and puromycin transcript from the derepressed *VSG221* ES after the induction of *SMC3* RNAi with tetracycline for the time indicated. E. Decrease in *VSGT3* transcript from the active *VSGT3* ES after knock-down of *SMC3* by the induction of *SMC3* RNAi. F. Increase in VSG switching after knock-down of SMC3 as analysed through monitoring *VSG* transcripts from silent *VSG* ESs as in (C).

In order to determine if the *VSG* ES promoter derepression observed after TbSpt16 knock-down results in processive transcription of silent *VSG* ESs, we monitored *VSG* transcripts from silent *VSG* ESs. The *T. brucei* RY-SP1 reporter line was grown in blasticidin to maintain a population homogeneous for expression of the active *VSGT3* ES. Cells were released from blasticidin selection for two generation times before TbSpt16 RNAi was induced. Total RNA was isolated, and transcript levels were monitored with qPCR (Hughes *et al*., 2007; Yang *et al*., 2009). As expected from the FACS analysis, there was a significant 15-fold increase in *eGFP* transcript (Fig. 8A). However, after the induction of TbSpt16 RNAi, we found a dramatic decrease in *VSGT3* transcript from the active *VSGT3* ES down to 35% original levels after 24 h and 0.2% original levels after 48 h (Fig. 8B). Knock-down of TbSpt16 for up to 48 h did not lead to a significant increase in *VSG* transcripts from six different silent *VSG* ESs (Fig. 8C). This indicates that although blocking synthesis of TbSpt16 results in *VSG* ES promoter derepression, transcription is not processive, and does not extend down to the telomere end resulting in full *VSG* ES activation.

Recently, the cohesin complex has been reported to be critical for monoallelic expression of *VSG* ESs (Landeira *et al*., 2009). Cohesin is a ring-like structure with four subunits: SMC1, SMC3, SCC1 and SCC3 which are involved in cohesion of sister chromatids. Partial knock-down of cohesin subunits results in an increase in *VSG* ES switching (Landeira *et al*., 2009), as well as disruption of chromosome segregation (Gluenz *et al*., 2008; Bessat and Ersfeld, 2009). To determine whether disruption of chromosome segregration invariably leads to significant *VSG* ES promoter derepression, we investigated the effects of TbSMC3 knock-down. Using the *T. brucei* RY-SM1 cell line, we showed that as expected knock-down of TbSMC3 led to a reduction in cell growth (Figs S4A and S4B). However, only minimal derepression of *VSG* ES promoters was observed (Figs 8D and S4C), with efficent knock-down of TbSMC3 protein after 48 h induction of TbSMC3 RNAi (Fig. S4D).

Depletion of the cohesin subunit TbSMC3 also led to a decrease in *VSG* transcript from the active *VSG* ES, although the kinetics were slower than after knock-down of TbSpt16 (Fig. 8E). Blocking cells in nuclear division possibly leads to disruption of the ES body containing the active *VSG* ES (Navarro and Gull, 2001), leading to problems with maintaining a fully transcriptionally active *VSG* ES (Landeira *et al*., 2009). Although downregulation of TbSMC3 led to only a small increase in *VSG* ES promoter proximal transcripts, *VSG* ES switching was increased (Fig. 8F) as also seen by Landeira *et al*. (2009). This result argues that the significant *VSG* ES promoter derepression observed after blocking TbSpt16 synthesis is not simply a consequence of disruption in chromosome segregation but a more direct effect of chromatin remodelling by FACT.

In order to investigate the effect of the knock-down of TbSpt16 on RNA polymerase II derived transcripts, we determined levels of γ-tubulin and actin transcripts after the induction of RNAi (Fig. S5A). In contrast to the observed dramatic decrease in transcript from the active *VSG*, there was only limited reduction in these Pol II transcripts to 50% normal levels after induction of TbSpt16 RNAi for 48 h. After the induction of TbSMC3 RNAi, only marginal decreases in these transcripts were observed (Fig. S5B). Transcripts from the spliced leader (SL) RNA genes did not change after knock-down of either TbSpt16 or SMC3.

### TbSpt16 is enriched on silent *VSG* ES promoters

**Fig. 9:**
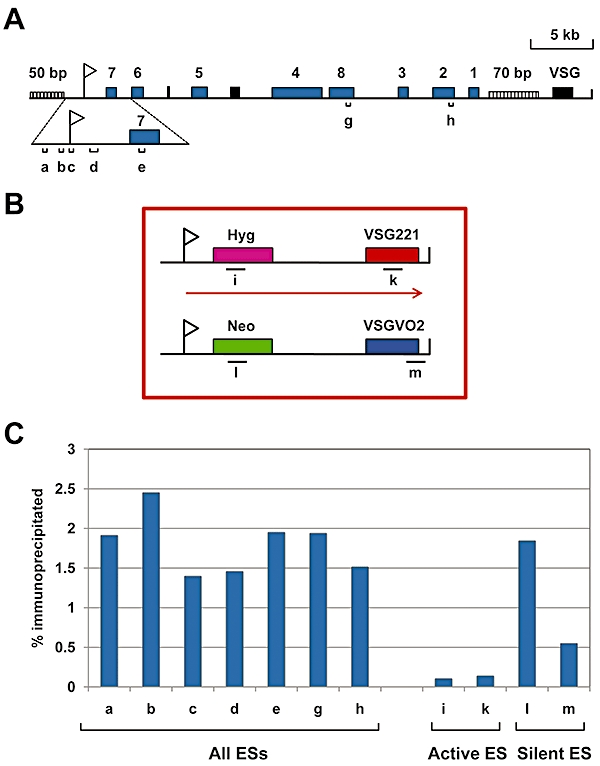
TbSpt16 binds *VSG* ESs, and is particularly enriched on the silent *VSG* ES promoter region. ChIP using myc-tagged TbSpt16 was performed as described in Stanne and Rudenko (2010), and all primer pairs used for qPCR are labelled according to Stanne and Rudenko (2010). A. A schematic of a typical *VSG* ES is shown, with the promoter indicated with a white flag, and different expression site associated genes with filled boxes. Characteristic 50 bp or 70 bp simple sequence repeats are indicated with vertically striped boxes. The primers indicated can be expected to recognize most if not all ESs. B. The *T. brucei* cell line used for TbSpt16 ChIP is shown. The active *VSG221* ES contains a hygromycin resistance gene immediately behind the ES promoter (Hyg). The silent *VSGVO2* ES contains a neomycin resistance gene (Neo) inserted behind the silent promoter. The primers indicated only recognize single-copy sequences (Stanne and Rudenko, 2010). C. Distribution of TbSpt16 along the *VSG* ES as investigated by ChIP and qPCR. The percentage of total input immunoprecipitated is plotted on the *y*-axis. The mean of two independent experiments is shown.

The derepression of silent ES promoters observed after the knock-down of TbSpt16 could indicate a direct role for TbSpt16 at the ES promoter itself. Alternatively, the observed ES derepression could be the consequence of an indirect effect operating on proteins downstream of TbSpt16. In order to distinguish between these possibilities, we therefore performed chromatin immunoprecipitation (ChIP) experiments in a cell line where TbSpt16 was tagged with a myc-epitope (Fig. 9). As shown in Fig. 9C, TbSpt16 binds the entire ES. However, the primer pairs used (primers a–h) could be expected to recognize most if not all ESs irrespective of their transcriptional state. Using single-copy marker genes allowing us to distinguish between active and silent ESs, we show that there is a striking enrichment of TbSpt16 at the promoter region of the silent *VSGVO2* ES. Relatively little TbSpt16 was found to bind the active *VSG221* ES (Fig. 9). These experiments provide supporting evidence for a direct role for TbSpt16 in ES silencing.

## Discussion

Here we show that inhibition of the TbSpt16 subunit of the FACT complex in *T. brucei* triggers a cell cycle arrest in the G2/early M cell cycle stage in both bloodstream and insect form *T. brucei*, with disruption of minichromosome segregation in both life cycle stages. Concurrent with this cell cycle arrest, disruption of TbSpt16 results in 20- to 23-fold derepression of *VSG* ESs in bloodstream form *T. brucei* and 16- to 25-fold derepression in procyclic *T. brucei*. Strikingly, this *VSG* ES derepression was strongly correlated with cells in the G2/early M phase of the cell cycle. We did not observe an increase in *VSG* ES switching in these stalled cells, indicating that derepressed *VSG* ES transcription was not fully processive. *VSG* ES promoter derepression was not simply a consequence of a disruption in chromosome segregation, as the induction of a block in synthesis of the SMC3 cohesin subunit did not lead to comparable levels of promoter derepression. ChIP experiments showed that TbSpt16 binds ESs, and is relatively enriched at silent but not active ES promoters. Our data are therefore compatible with FACT playing a role in the maintenance of the repressed chromatin state present at the inactive *VSG* ES promoters. The failure of cells to properly partition chromosomes in the absence of TbSpt16 also indicates an involvement of FACT in the maintenance of a chromatin status allowing their mitotic segregation. We hypothesize that the FACT complex plays a role in establishing or maintaining the heterochromatin status of centromeric sequences.

In other organisms the FACT complex has been shown to function as a histone chaperone. Functions for FACT include facilitating transcription elongation and the maintenance of heterochromatin necessary for suppression of inappropriate transcription, as well as facilitating DNA replication and the maintenance of functional centromeres (Reinberg and Sims 3rd, 2006; Tan *et al*., 2006; Formosa, 2008; Park and Luger, 2008). FACT facilitates chromatin transcription by all three nuclear RNA polymerases (Belotserkovskaya *et al*., 2003; Birch *et al*., 2009) by destabilizing the nucleosomal structure (Belotserkovskaya *et al*., 2003; Stuwe *et al*., 2008). This opens the DNA template for transcription, and facilitates the transcribing RNA polymerase. In addition, FACT reassembles nucleosomes and restores chromatin structure after passage of the elongating RNA polymerase. This activity results in the repression of fortuitous initiation of transcription from regions, which would otherwise be transcribed from cryptic promoters (Kaplan *et al*., 2003; Mason and Struhl, 2003). Lastly, FACT plays a role in the maintenance of functional centromeric heterochromatin (Lejeune *et al*., 2007; Okada *et al*., 2009).

We observe in *T. brucei* that knock-down of the TbSpt16 subunit of FACT triggers a precise cell cycle arrest in G2/early M, with cells containing a 4n DNA content compatible with completed DNA replication and exit from S phase. There was however a clear disruption in segregation of the *T. brucei* minichromosomes. This suggests that in the absence of FACT, centromeric function in this minichromosomal size class is compromised. *T. brucei* minichromosomes segregate before *T. brucei* large chromosomes through a different segregation mechanism (Ersfeld and Gull, 1997; Gull *et al*., 1998). The centromeres of the large chromosomes of *T. brucei* have been mapped via cleavage by topoisomerase II (Obado *et al*., 2007), but the centromeres of minichromosomes have not been defined. They are however likely to involve a particular chromatin state of the palindromic arrays of 177 bp simple sequence repeats, which comprise the bulk of the *T. brucei* minichromosomes (Gull *et al*., 1998; Wickstead *et al*., 2004). It is likely that blocking synthesis of TbSpt16 impacts on segregation of the large chromosomes as well as the minichromosomes, especially as recent reports show the importance of FACT mediated centromeric chromatin structure in other eukaryotes (Lejeune *et al*., 2007; Okada *et al*., 2009). However, as our cells arrest in G2/early M, and do not progress through the cell cycle to anaphase, it was not possible for us to investigate if TbSpt16 knock-down also triggers disruption in segregation of the *T. brucei* large chromosomes.

We found that blocking synthesis of TbSpt16 results in high levels of derepression of *VSG* ESs in both bloodstream and procyclic form *T. brucei* comparable to what had been observed after knock-down of the SWI2/SNF2 chromatin remodeler TbISWI (Hughes *et al*., 2007). As discussed earlier (Hughes *et al*., 2007), although these levels of ES derepression are striking, they are only about 10% of the level of expression from an active ES. Possibly, maximal activation of all 15 ESs is not possible as one or more essential transcription factors become limiting. This ES derepression is not likely to be mechanistically similar to as observed after the induction of DNA damage in *T. brucei*, as cells where TbSpt16 synthesis is blocked arrest at G2/M rather than the G1/S phase (Sheader *et al*., 2004).

In *S. cerevisiae* FACT plays a role in maintaining the chromatin structure, which mediates suppression of transcription from cryptic promoters (Kaplan *et al*., 2003; Mason and Struhl, 2003). In bloodstream form *T. brucei* silent *VSG* ESs are relatively enriched in nucleosomes compared with the active *VSG* ES (Figueiredo and Cross, 2010; Stanne and Rudenko, 2010). Despite the relatively repressed chromatin state, silent *VSG* ES promoters are transcriptionally active in both bloodstream and procyclic form *T. brucei*, but transcription elongation is impaired in all but the ‘active’ bloodstream form *VSG* ES (Vanhamme *et al*., 2000). TbSpt16 is found to be particularly enriched at the promoter region of silent ESs, but does not appear to be abundant on the active ES. It is therefore possible that in *T. brucei* FACT plays a role in the maintenance of the repressed chromatin structure present at these silent *VSG* ES promoters, and that in the absence of TbSpt16 abortive transcripts from the inactive *VSG* ES promoters become more abundant.

A striking feature of the derepression of *VSG* ES promoters seen after a TbSpt16 synthesis block is that it is observed primarily in cells stalled in G2/early M. Possibly, FACT is particularly relevant for chromatin maintenance in *T. brucei* immediately after DNA replication. Although significant *VSG* ES promoter derepression was seen after blocking TbSpt16 synthesis, there was no large increase in transcript levels from silent *VSG* ESs, indicating that fully processive transcription down to the telomere end was not increased. As disruption of cohesin subunits has been proposed to result in an increase in *VSG* ES switching (Landeira *et al*., 2009), we reinvestigated this as a control. Our results are in agreement. We observed only minor derepression of *VSG* ES promoters after an *SMC3* knock-down, but an increase in *VSG* ES switching. These complementing results argue that the observed *VSG* ES promoter derepression after a TbSpt16 knock-down is not simply a consequence of the induction of a cell cycle arrest in G2/early M, but is due to specific chromatin remodelling activity of FACT during this stage of the cell cycle. Landeira *et al*. argue that the maintenance of sister chromatid cohesion ensures monallelic expression of *VSG* during nuclear division. Our results are compatible with this, as we see a reduction in transcription from the active *VSG* ES after knocking down either TbSpt16 or SMC3.

We do not observe an increase in *VSG* ES switching after the induction of TbSpt16 RNAi, but these results are complicated by the observation that *VSG* transcript from the active *VSG* ES is dramatically reduced down to 0.2% original levels after 48 h induction of TbSpt16 RNAi. It is therefore possible that FACT also plays a role in processive Pol I transcription of *VSG* ESs. As processive transcription of the active *VSG* ES appears to be compromised in our cells, it cannot be excluded that this is also the case with transcription derived from silent *VSG* ESs.

Although knock-down of TbSpt16 transcript for 48 h resulted in a dramatic decrease in *VSG* transcript from the active Pol I transcribed *VSG* ES, only a relatively minor reduction in the RNA Pol II derived transcripts γ-tubulin, actin and SL was observed. In other organisms FACT has been shown to facilitate the elongation of RNA polymerase II transcription (Belotserkovskaya *et al*., 2003; Mason and Struhl, 2003; Saunders *et al*., 2003). One scenario explaining the lack of a prominent effect on Pol II transcripts observed in *T. brucei* could be that RNA polymerase II transcription in *T. brucei* is not transcriptionally regulated, unlike in the organisms (including *S. cerevisiae* and mammals), where FACT has been extensively investigated. As most of the *T. brucei* genome is constitutively transcribed in polycistronic arrays, there could be less need to use chromatin structure to suppress fortuitous initiation at cryptic promoters. It is therefore possible that in *T. brucei* there is only a relatively minor role for FACT in re-establishing nucleosomes after the elongating RNA polymerase II. In contrast, in the regulated RNA pol I transcribed *VSG* ESs of *T. brucei* there could be a greater need for a chromatin remodelling activity as the cell needs to reset different chromatin states during the life cycle.

In addition to its role in transcriptional control, the FACT complex has been shown to be functionally linked to DNA replication in mammalian cells, and is thought to facilitate chromatin unwinding by the MCM complex (Tan *et al*., 2006). We did not find any clear evidence that blocking synthesis of TbSpt16 leads to a disruption of S phase, as the stalled cells had a 4n DNA content compatible with unimpaired DNA replication and successful completion of S phase.

Our results further extend the observation that chromatin remodelling is critical for *VSG* ES control in *T. brucei* (Figueiredo and Cross, 2010; Stanne and Rudenko, 2010). ‘Silent’*VSG* ES promoters are transcribed at a low rate by non-processive RNA polymerase I (Vanhamme *et al*., 2000). *T. brucei* FACT presumably contributes to the downregulation of ‘silent’*VSG* ES promoters by remodelling nucleosomes and ‘re-silencing’ the region immediately around the extending RNA polymerase I molecules. However, FACT provides only one of the many layers of *VSG* ES control. The ATP-dependent chromatin remodeler TbISWI is involved in maintenance of repressed chromatin at silent *VSG* ESs, the telomere binding protein TbRAP1 maintains a silencing gradient operating from and extending upwards from the *VSG* ES telomere end, and the histone methyltransferase DOT1B is necessary for the maintenance of strict *VSG* ES silencing (Hughes *et al*., 2007; Figueiredo *et al*., 2008; Horn, 2009; Yang *et al*., 2009). Further studies will be needed to better characterize the different epigenetic modifications that distinguish the chromatin of the active *VSG* ES from the many silent ones. The challenge will come in trying to understand the synergies and hierarchies between these different chromatin remodelling activities, and how these all contribute to the observed *VSG* ES control.

## Experimental procedures

### Trypanosome cell lines and cultivation

Bloodstream form *T. brucei* 427 was cultured in HMI-9 medium containing 20% foetal calf serum (FCS) at 37°C using the appropriate drugs (Hirumi and Hirumi, 1989). The parental bloodstream form *T. brucei* line RY was the VSGT3-expressing *T. brucei* T3-SM cell line (Hughes *et al*., 2007). A blasticidin resistance gene located downstream of the active *VSGT3* ES promoter allowed for selection of a homogeneous population of VSGT3 expressors, and an *eGFP* gene downstream of the silent *VSG221* ES promoter allowed for monitoring of *VSG* ES derepression. The *T. brucei* RY-SP1 line contains a TbSpt16 RNAi construct with opposing T7 promoters inserted into *T. brucei* minichromosomes (Wickstead *et al*., 2002). The sequence of the TbSpt16 RNAi fragment is GeneDB: Tb927.3.5620 position 1408–2185. The *T. brucei* RY-SM1 line is comparable, but has aTbSMC3 RNAi construct, where the TbSMC3 sequence used in the vector is GeneDB: Tb927.5.3510, position 1978–2694. The same TbSpt16 RNAi construct was also transfected into the single marker cell line (Wirtz *et al*., 1999). The TbISWI RNAi cell line is described in Hughes *et al*. (2007).

Procyclic form *T. brucei* was cultured in SDM-79 medium at 27°C containing 10% FCS, 5 mg l^−1^ haemin, and relevant drugs. RNAi constructs were either integrated into the *T. brucei* 29–13 cell line (Wirtz *et al*., 1999) (PF-SP1 and PF-SP2) or the *T. brucei* D3 reporter cell line (D3-SP1 and D3-SP2) (Hughes *et al*., 2007). Growth curves were performed by culturing cells and determining cell density until the parental cell line reached stationary phase.

### Sequence analysis

Protein sequence alignments were performed using ClustalW2. TbSpt16 (GeneDB: Tb927.3.5620) was compared with orthologues in *Saccharomyces cerevisiae* (SGD: YGL207W), *Schizosaccharomyces pombe* (GeneDB: SPBP8B7.19), *Caenorhabditis elegans* (Wormbase: Q9N5R9), *Drosophila melanogaster* (PubMed: Q8IRG6), *Mus musculus* (PubMed: Q920B9) and *Homo sapiens* (PubMed: Q9Y5B9). The three annotated domains using SMART (aminopeptidase domain, catalytic core PF08644 and histone chaperone domain PF08512) were aligned separately.

### Protein analysis

Protein lysates were made essentially according to Hughes *et al*. (2007) with the exception that the centrifugation step for lysate fractionation was eliminated, and total lysate was used. Western blot analysis was performed using standard procedures with an anti-SMC3 antibody (Bessat and Ersfeld, 2009) or anti-Spt16 antibody. The anti-TbSpt16 antibody is a polyclonal antibody made in rabbits immunized with a segment of TbSpt16 (position 319–993 of the open reading frame) fused to a 6× His tag (Invitrogen). The antibody against BiP was a gift of Jay Bangs.

### Flow cytometry

In order to investigate *T. brucei VSG* ES derepression, *T. brucei* cultures were washed, derepression of *eGFP* was monitored in the FL-1 channel, and derepression of *DsRed* was monitored in the FL-2 channel of a Beckman FACSCalibur (BD Biosciences). Cell cycle analysis was performed with PI stained cells. Bloodstream form *T. brucei* was washed, fixed in 2% paraformaldehyde, washed, permeabilized at room temperature for 10 min with 0.1% Triton and then washed again. Cells were subsequently resuspended in PBS containing 10 µg ml^−1^ RNase A and 10 µg ml^−1^ PI. After incubating for 45 min at 37°C, PI staining was monitored by FACS in the FL-2 channel, and *eGFP* expression was simultaneously monitored in the FL-1 channel. Cell cycle analysis of procyclic form *T. brucei* was performed according to Bessat and Ersfeld (2009).

### Fluorescent in situ hybridization

Fluorescent *in situ* hybridization experiments were performed as in Bessat and Ersfeld (2009). In order to permeabilize the cells after fixation with formaldehyde, bloodstream form *T. brucei* was incubated for 10 min and procyclic form *T. brucei* for 5 min with 0.1% NP40 in PBS. Samples were analysed by fluorescence microscopy using a Zeiss Axioplan 2 microscope and a Roper CoolSnap HQ camera. Each experiment was performed twice, and approximately 400 cells were counted per experimental sample.

### Monitoring of DNA replication

DNA replication was monitored using microscopic analysis of cells where BrdU was incorporated, and visualized using an anti-BrdU antibody. Cells were incubated for 1 h with 50 µM BrdU (Sigma, stock solution 5 mM in water). Immunofluorescence microscopy was performed as modified from Woodward and Gull (1990). Cells were incubated with an anti-BrdU antibody (Molecular Probes) before analysis by fluorescence microscopy. At least 300 cells were analysed per sample.

### qPCR quantification of RNA transcripts

The *T. brucei* RY-SP1 and *T. brucei* RY-SM1 cell lines were removed from blasticidin selection for 20 h (about two generation times) in order to remove selection pressure for maintenance of the *VSGT3* ES, and allow switching to other *VSG* ESs. After induction of RNAi, total RNA was isolated using the RNeasy kit (Qiagen). RNA was subsequently DNase treated using the TURBO DNA-free kit (Ambion). 1 µg RNA per sample was used as a template for cDNA synthesis using the Omniscript RT kit (Qiagen). qPCR was performed using SYBR Green I Master (Roche Applied Science) and detected via a LightCycler 480 (Roche Diagnostics). Reaction conditions were optimized for each primer pair individually. Non-reverse transcribed RNA served as a negative control for gDNA contamination. Each experiment was performed in triplicate.

### Chromatin immunoprecipitation

TbSpt16 was tagged with a C-terminal myc epitope by transfecting *T. brucei* HNI(221+) (Rudenko *et al*., 1998) with the pMoTag43MB Spt16 construct (Oberholzer *et al*., 2006) to generate *T. brucei* myc-Spt16. This cell line has an active *VSG221* ES and a silent *VSGVO2* ES. ChIP was performed essentially as described previously (Lowell and Cross, 2004; Stanne and Rudenko, 2010); only cross-linking was performed using 1% formaldehyde for 60 min at room temperature. For immunoprecipitation of myc-Spt16 a monoclonal anti-myc antibody (clone 9E10, Sigma) was used. Quantitative PCR (qPCR) was performed as described in Stanne and Rudenko (2010). ChIP of histone H3 was performed as a control (data not shown). Data presented are the mean of two independent ChIP experiments.
