# Supplementary material for: The FACT subunit TbSpt16 is involved in cell cycle specific control of VSG expression sites in Trypanosoma brucei
Source: Mol Microbiol. 2010 Oct;78(2):459–74. doi: 10.1111/j.1365-2958.2010.07350.x (PMC3034197; doi:10.1111/j.1365-2958.2010.07350.x)
Supplement: Supplementary file 1 [file mmi0078-0459-SD1.pdf]

Supplementary materials for:

The FACT subunit TbSpt16 is involved in cell cycle specific control of *VSG* expression sites in *Trypanosoma brucei* *by*

Viola Denninger[1], Alexander Fullbrook[1], Mohamed Bessat[2], Klaus Ersfeld[2] and Gloria Rudenko[1*]

[1]Division of Cell and Molecular Biology, Sir Alexander Fleming Building, Imperial College, South Kensington, London SW7 2AZ, UK; [2]Department of Biological Sciences and Hull York Medical School, University of Hull, Cottingham Road, Hull HU6 7RX, UK.

Correspondence should be addressed to:

Dr. Gloria Rudenko

E-mail: gloria.rudenko@imperial.ac.uk

Tel: +44 207 594 8137

FAX: +44 207 584 2056

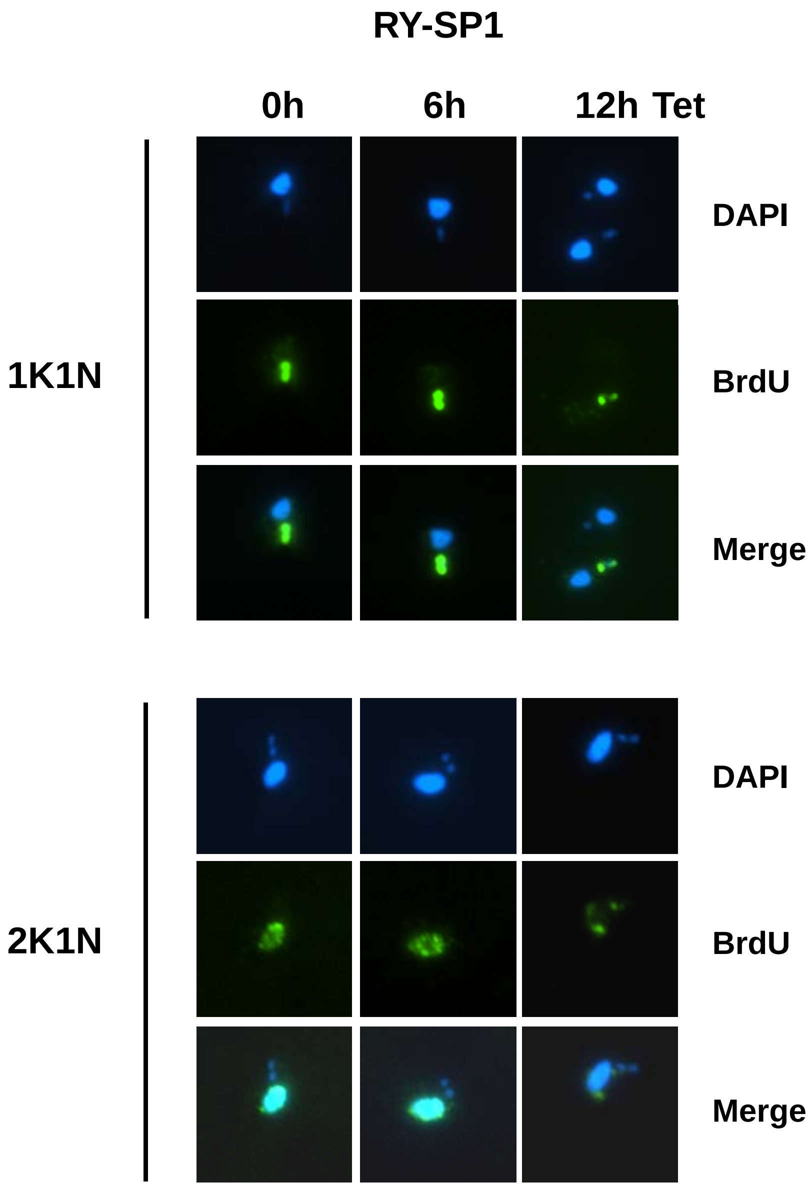

Fig. S1

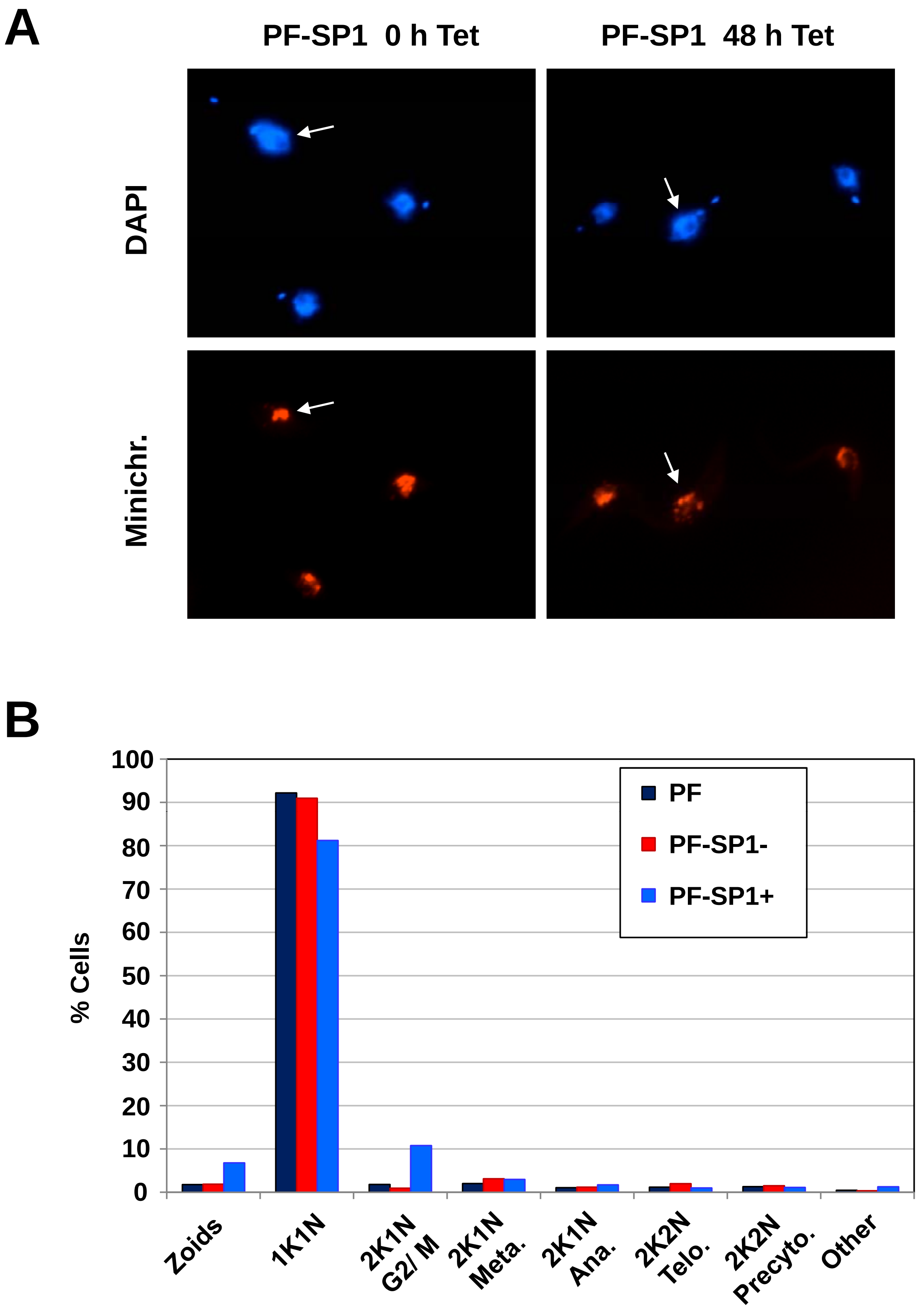
A
PF-SP1 0 h Tet
PF-SP1 48 h Tet
DAPI
Minichr.
B
100
90
80
70
60
50
40
30
20
10
0
% Cells
PF
PF-SP1-
PF-SP1+
Zoids
1K1N
2K1N G2/ M
2K1N Meta.
2K1N Ana.
2K2N Telo.
2K2N Precyto.
Other

Fig. S2

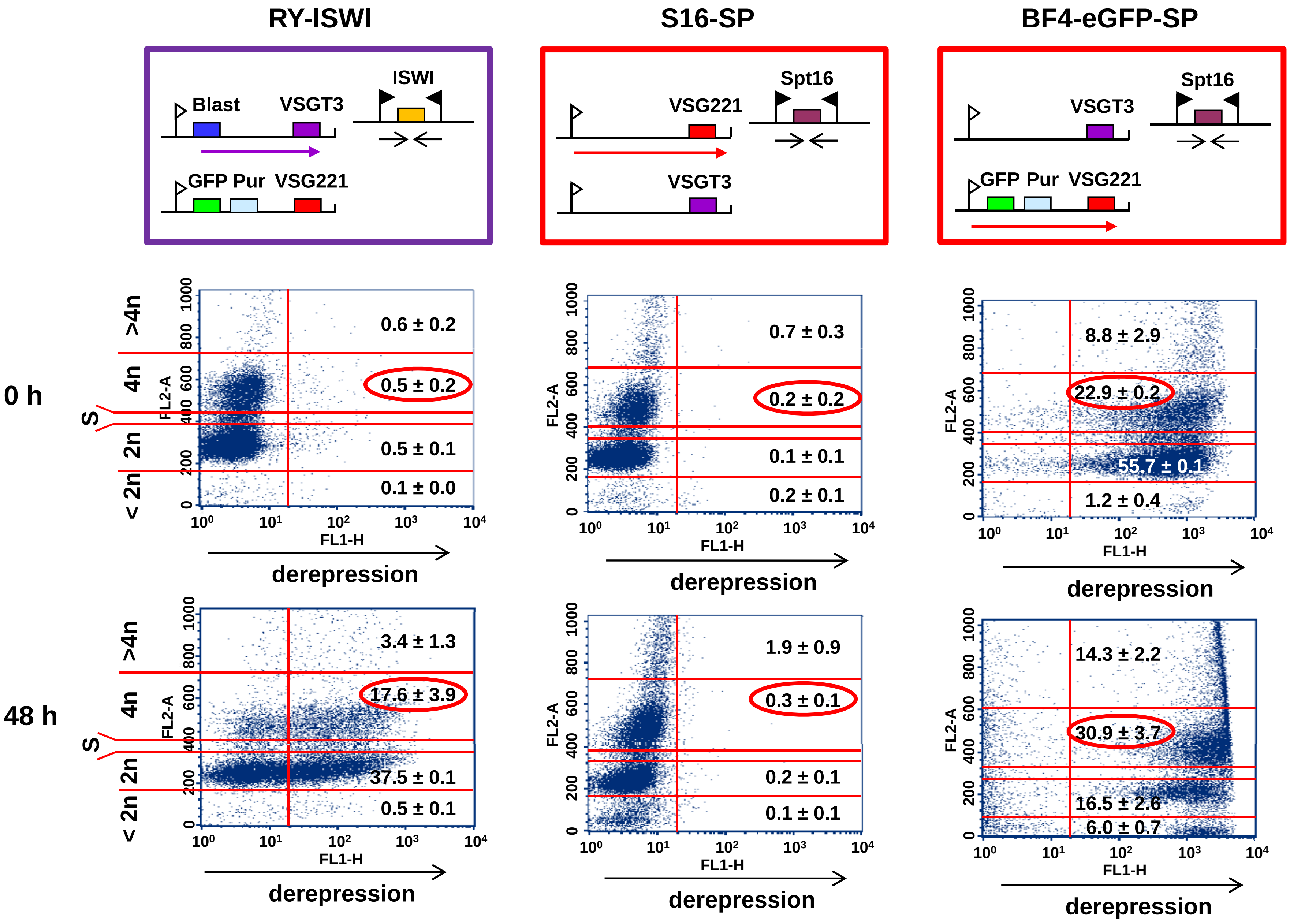

RY-ISWI
S16-SP
BF4-eGFP-SP
Blast
VSGT3
ISWI
GFP Pur VSG221
VSG221
Spt16
VSGT3
VSGT3
Spt16
GFP Pur VSG221
0 h
48 h
>4n
4n
S
2n
< 2n
FL2-A
FL1-H
derepression
0.6 ± 0.2
0.5 ± 0.2
0.5 ± 0.1
0.1 ± 0.0
0.7 ± 0.3
0.2 ± 0.2
0.1 ± 0.1
0.2 ± 0.1
8.8 ± 2.9
22.9 ± 0.2
55.7 ± 0.1
1.2 ± 0.4
3.4 ± 1.3
17.6 ± 3.9
37.5 ± 0.1
0.5 ± 0.1
1.9 ± 0.9
0.3 ± 0.1
0.2 ± 0.1
0.1 ± 0.1
14.3 ± 2.2
30.9 ± 3.7
16.5 ± 2.6
6.0 ± 0.7

Fig. S3

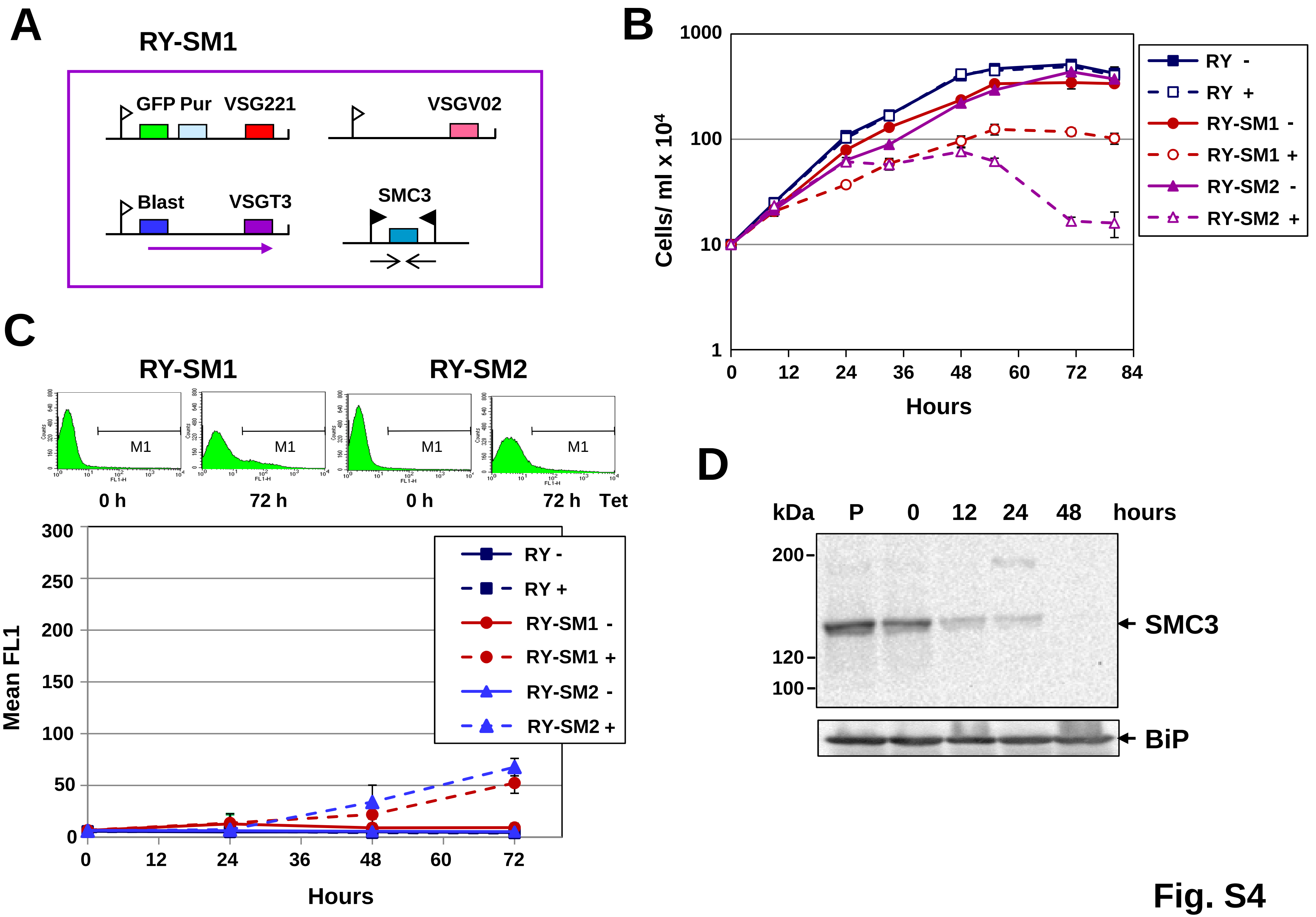
A
RY-SM1
GFP Pur VSG221
VSGV02
Blast
VSGT3
SMC3
B
1000
100
10
1
Cells/ ml x 10^4
0 12 24 36 48 60 72 84
Hours
RY -
RY +
RY-SM1 -
RY-SM1 +
RY-SM2 -
RY-SM2 +
C
RY-SM1
RY-SM2
M1
0 h
72 h
0 h
72 h
Tet
Mean FL1
300
250
200
150
100
50
0
0 12 24 36 48 60 72
Hours
RY -
RY +
RY-SM1 -
RY-SM1 +
RY-SM2 -
RY-SM2 +
D
kDa P 0 12 24 48 hours
200
120
100
SMC3
BiP

Fig. S4

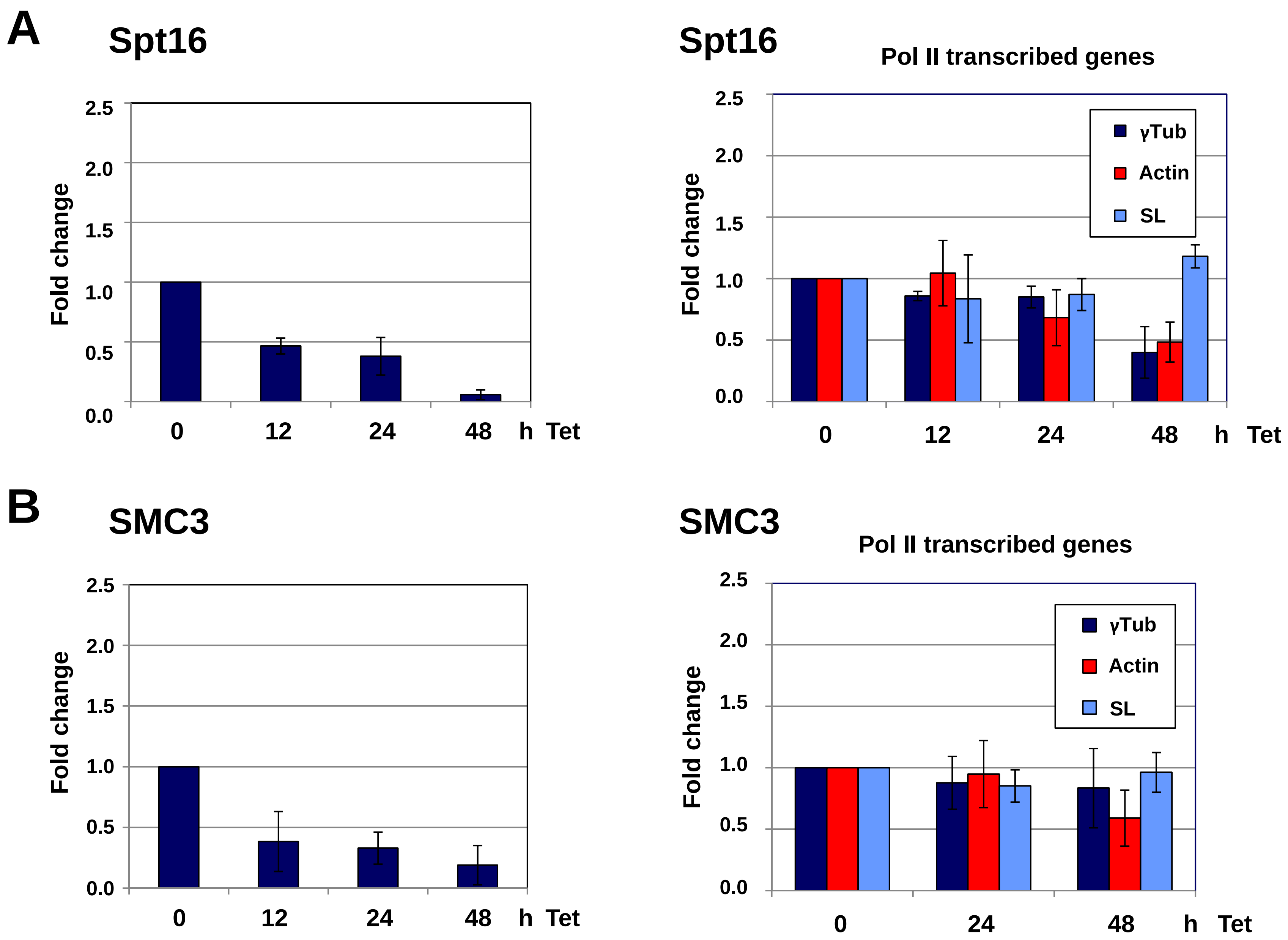

A
Spt16
2.5
2.0
1.5
1.0
0.5
0.0
Fold change
0
12
24
48
h Tet
Spt16
Pol II transcribed genes
γTub
Actin
SL
B
SMC3
SMC3
Pol II transcribed genes

Fig. S5

Supplementary Figure legends

Fig. S1

DNA replication in cells where TbSpt16 synthesis has been blocked. Bloodstream form *T. brucei* RY-SP1 was incubated with tetracycline (Tet) to induce TbSpt16 RNAi for the time in hours (h) indicated above. Following tetracycline induction, cells were incubated with 5-bromo-2'-deoxyuridine (BrdU) for one hour, allowing DNA replication in the form of BrdU incorporation to be visualised by immunofluorescence using an anti-BrdU antibody. Representative 1K1N cells (where replication is predominantly found in the kinetoplast and the newly replicated DNA is found at the poles of the kinetoplast) and 2K1N cells (where replication and division of the kinetoplast is already finished and only replication of the nuclear DNA is going on) are shown, with panels showing DAPI stained cells, cells where incorporated BrdU has been visualised by immunofluroescence (BrdU), and a merge of the two images.

Fig. S2

Disruption of minichromosome segregation in insect form *T. brucei* after blocking TbSpt16 synthesis.

(**A**) Procyclic form *T. brucei* PF-SP1 was incubated with tetracycline (Tet) to induce TbSpt16 RNAi for the time in hours (h) indicated above. Fluorescent in situ hybridisation (FISH) was performed using a 177 bp repeat probe which is specific for minichromosomes (Minichr.). The top panels show DAPI stained cells. 2K1N cells are indicated with arrows. Similar to as observed in bloodstream form *T. brucei,* after the induction of TbSpt16 RNAi, minichromosomes do not cluster at metaphase, but appear dispersed throughout the cell indicating disruption of chromosome segregation.

(**B**) Accumulation of cells in G2/ M and zoids after the induction of TbSpt16 RNAi for 48 hours. Quantitation of a representative experiment, with the percentage of cells in the different cell cycle categories (see Fig. 3A) plotted.

Fig. S3

Depletion of TbSpt16 leads to a cell cycle specific derepression of silent *VSG* ESs. Above are schematics of the *T. brucei* cell lines analysed. *T. brucei* RY-ISWI is described in (Hughes *et al.*, 2007) and contains a TbISWI RNAi construct. *T. brucei* S16-SP is the single marker cell line (Wirtz *et al.*, 1999) that has been transfected with the TbSpt16 RNAi construct. *T. brucei* BF4-eGFP-SP expresses eGFP from the active *VSG221* ES, and has been transfected with the TbSpt16 RNAi construct. The ES promoters are indicated with white flags, the inducible T7 promoters on the RNAi constructs are indicated with black flags, and the filled boxes indicate various genes including blasticidin resistance (Blast), GFP and puromycin resistance (Pur). Transcription is indicated with arrows.

Below representative FACS traces are shown before (0 hours) or after 48 hours of tetracycline induction of TbISWI or TbSpt16 RNAi. DNA content of propidium iodide stained cells as monitored in the FL2 channel is plotted on the Y-axis, with the horizontal red lines subdividing populations with DNA contents of respectively <2n, 2n, S, 4n or >4n. Derepression of eGFP as monitored in the FL1 channel is plotted on the X-axis. Cells with levels of eGFP expression above 20 were considered significantly derepressed, and are to the right of the red line. The percentage of total cells present in different boxes is indicated, with particularly relevant values highlighted with a red oval. A representative experiment for each cell line is

shown, with the values presented being the mean of three independent experiments with the standard deviation indicated.

Fig. S4

Downregulation of the cohesin subunit SMC3 leads to a growth arrest in bloodstream form *T. brucei* but minimal derepression of *VSG* ESs.

(**A**) Schematic of the bloodstream form *T. brucei* reporter line RY-SM1. The large violet box indicates the trypanosome, with a blasticidin resistance gene (Blast) inserted in the active *VSGT3* ES. *VSG* ES promoters are indicated with white flags, and ES transcription with a violet arrow. The inactive *VSG221* ES has *eGFP* and a puromycin resistance gene (Pur) inserted immediately downstream of the promoter. TbSMC3 RNAi is induced from opposing tetracycline inducible T7 promoters (black flags). Derepression of *eGFP* can be monitored by FACS.

(**B**) Growth curve of two independent bloodstream form *T. brucei* clones RY-SM1 and RY-SM2 grown in the presence (+) or absence (-) of tetracycline for the time in hours indicated to induce RNAi against TbSMC3. The parental line RY is shown in comparison.

(**C**) Blocking synthesis of TbSMC3 by the induction of RNAi against TbSMC3 results in minimal derepression of *VSG* ESs in bloodstream form *T. brucei*. *T. brucei* RY-SM1 and RY-SM2 cells were incubated in the presence (+) or absence (-) of tetracycline to induce TbSMC3 RNAi, and cells were monitored for fluorescence in the FL1 channel. Cell fluorescence indicates derepression of the *eGFP* gene present in the silent *VSG221* ES. Representative FACS traces are shown of cells incubated in the presence or absence of tetracycline (Tet) for the time indicated in hours (h). The graph shows the total degree of fluorescence in the FL1 channel plotted over

time. The results shown are the mean of three independent experiments with the standard deviation indicated with error bars.

(**D**) Successful knockdown of TbSMC3 as shown by Western blotting. Protein lysates were isolated from *T. brucei* RY-SM1 after RNAi against TbSMC3 was induced with tetracycline for the time indicated in hours. Lysate from the parental cell line (P) is shown as a control. On the left are size markers with sizes indicated in kiloDaltons (kDa). The TbSMC3 band is indicated with an arrow. The blot was probed with anti-BiP antibody as a loading control.

Fig. S5

Transcript levels after the induction of TbSMC3 or TbSpt16 RNAi. RNAi was induced with tetracycline (Tet) for the time in hours (h) indicated. The level of transcript in the absence of RNAi is set at one, and the fold change is plotted over time. Results are the mean values of three independent experiments with the standard deviation indicated with error bars.

(**A**) On the left is shown knock-down of TbSpt16 transcript after the induction of TbSpt16 RNAi. On the right is shown quantitation of different RNA polymerase II derived transcripts (γ-tubulin, actin and the spliced leader (SL) RNA) after the induction of TbSpt16 RNAi for the time indicated.

(**B**) On the left is shown TbSMC3 transcript knock down after the induction of TbSMC3 RNAi. On the right is shown quantitation of three representative RNA polymerase II transcribed transcipts after the induction of TbSMC3 RNAi for the time indicated in hours. The transcripts shown are γ-tubulin (γ Tub), actin and the spliced leader (SL) RNA.

References

Hughes, K., M. Wand, L. Foulston, R. Young, K. Harley, S. Terry, K. Ersfeld & G. Rudenko, (2007) A novel ISWI is involved in VSG expression site downregulation in African trypanosomes. *Embo J* **26**: 2400-2410.

Wirtz, E., S. Leal, C. Ochatt & G. A. Cross, (1999) A tightly regulated inducible expression system for conditional gene knock-outs and dominant-negative genetics in Trypanosoma brucei. *Mol Biochem Parasitol* **99**: 89-101.
